# Breeding Ecology of the Critically Endangered Baer's Pochard (*Aythya baeri*): Nest Threats and Conservation Implications

**DOI:** 10.1002/ece3.73181

**Published:** 2026-02-27

**Authors:** Fuguang Ma, Yuanxing Ye, Lu Li, Siyu Geng, Zhongxin Li, Jianying Ren, Changqing Ding

**Affiliations:** ^1^ School of Ecology and Nature Conservation Beijing Forestry University Beijing P. R. China; ^2^ Management Center of Minquan Yellow River Gudao National Wetland Park Henan P. R. China

**Keywords:** nest threats, reproduction success, waterbird conservation

## Abstract

Baer's Pochard ( *Aythya baeri* ), a diving duck endemic to eastern Asia, has once experienced a significant population decline and was classified as “Critically Endangered” in 2012. However, the factors contributing to this decline remain poorly understood. Nest survival is a crucial demographic parameter that significantly influences avian population dynamics and is often correlated with various environmental factors and the breeding period. We conducted research on its breeding biology from 2019 to 2022, documenting a total of 108 nests with laying dates ranging from 25 April to 15 July. The nest success rate was 24.97%. Predation was the primary cause of nest failure, accounting for 44.66% of nest failures, followed by desertion (17.48%), flooding (7.77%), and egg collecting by local people (6.80%). The analysis of the factors affecting daily nest survival indicated that daily nest survival was positively correlated with the height above water and the distance to shore of mound where they nested, but negatively correlated with the laying date. We suggest that controlling nest predation, providing suitable nesting sites, and maintaining suitable water levels are crucial for ensuring the nesting success of Baer's Pochard.

## Graphical abstract

This study identifies that the daily nest survival of Baer's Pochard was primarily influenced by three factors: it increased with greater distance from the shore and the height above water, and decreased with later laying dates. Both nest location and timing were identified as critical factors for reproductive success.

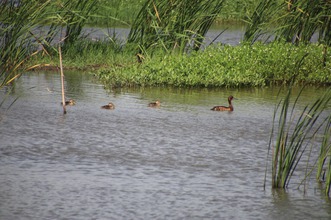

## 1 Introduction

**FIGURE 1:**
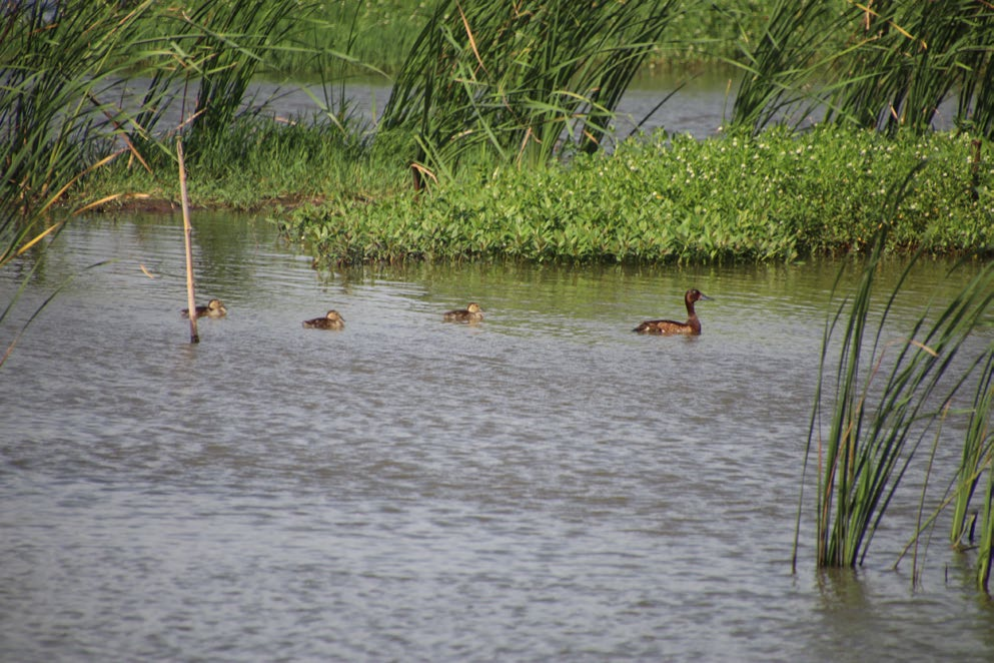
A female Baer's Pochard ( *Aythya baeri* ) with ducklings, photograph by Fuguang Ma in Jiujiang city, Jiangxi province.

The Baer's Pochard ( *Aythya baeri* , Figure 1) is a diving duck native to East Asia, which historically breeds in southeastern Russia and northeastern China (BirdLife International 2024). The Baer's Pochard was once a common and abundant migratory waterbird in the East Asian‐Australasia Flyway (Wang et al. 2012). After a sharp decline since the 1980s, the Baer's Pochard was classified as “critically endangered” by the IUCN in 2012 (BirdLife International 2012). Hearn et al. (2013) revealed that the population decline is primarily attributed to habitat loss and degradation, unsustainable harvesting, human disturbance, and a low reproductive success rate.

Recent studies have highlighted reproductive success as the most direct and important driving factor of water bird population dynamics (Koons et al. 2017; Rotelli et al. 2021; Pollentier et al. 2021; Weegman et al. 2022). Many studies have reported the link between breeding time and reproductive success (Ringelman et al. 2018; Setash et al. 2020). There are two primary hypotheses proposed to explain seasonal variation in reproductive success: the quality hypothesis and the date hypothesis. The former posits that early breeding individuals generally have better phenotypic quality and occupy more suitable habitats compared to those who breed late (Price et al. 1988; Verhulst and Tinbergen 1991). The latter suggests that the decline in reproductive success during the season is a result of the seasonal deterioration in environmental quality (Verhulst and Nilsson 2008).

Furthermore, nest habitat significantly influences nest survival. Increased vegetation coverage, water depth, distance to shore, and higher nest density may enhance nest success by complicating access for mammalian predators at a local scale (Austin and Buhl 2011; Bell and Conover 2023; Rime et al. 2024), as predation is the primary threat to nest survival, especially for ground‐nesting bird species (Smith et al. 2010). Other factors, including the areas and spatial concentration of wetlands in the surrounding landscape (Walker et al. 2013; Ringelman et al. 2018), landscape context, proximity to habitat edges (Shake et al. 2011), and access to high‐quality foraging sites (Holopainen et al. 2015), have also been found to affect nest success.

For diving ducks, nest distance to water has been reported to have a positive relationship with nest success (Walker et al. 2005; Navarre et al. 2022) due to their preference for nesting close to water to minimize overland travel for ducklings and reduce predation risks (Holopainen et al. 2015). However, nesting close to water could face the challenge of flooding, posing a significant threat to nest success in diving duck species (McAuley and Longcore 1989). At the same time, some studies have indicated that environmental factors may not significantly influence nest success (Maxson and Riggs 1996; O'Neil et al. 2014), suggesting complicated relationships between environment and nest success.

The Baer's Pochard remains a poorly studied species, primarily due to the challenges associated with surveying its distribution and habitat. A few studies have described the habitat use and behavior of Baer's Pochard (Wei et al. 2020; Li et al. 2024), but factors influencing nest survival remain poorly understood. This study aims to investigate reproductive parameters (timing of breeding, hatch rate, clutch size, and brood success) and breeding threats to Baer's Pochard. We also assess the impact of nesting time and habitat characteristics on daily nest survival (DNS), based on which we provide recommendations for conservation strategies and management practices during the nesting stage.

## 2 Methods

### 2.1 Study Area

**FIGURE 2:**
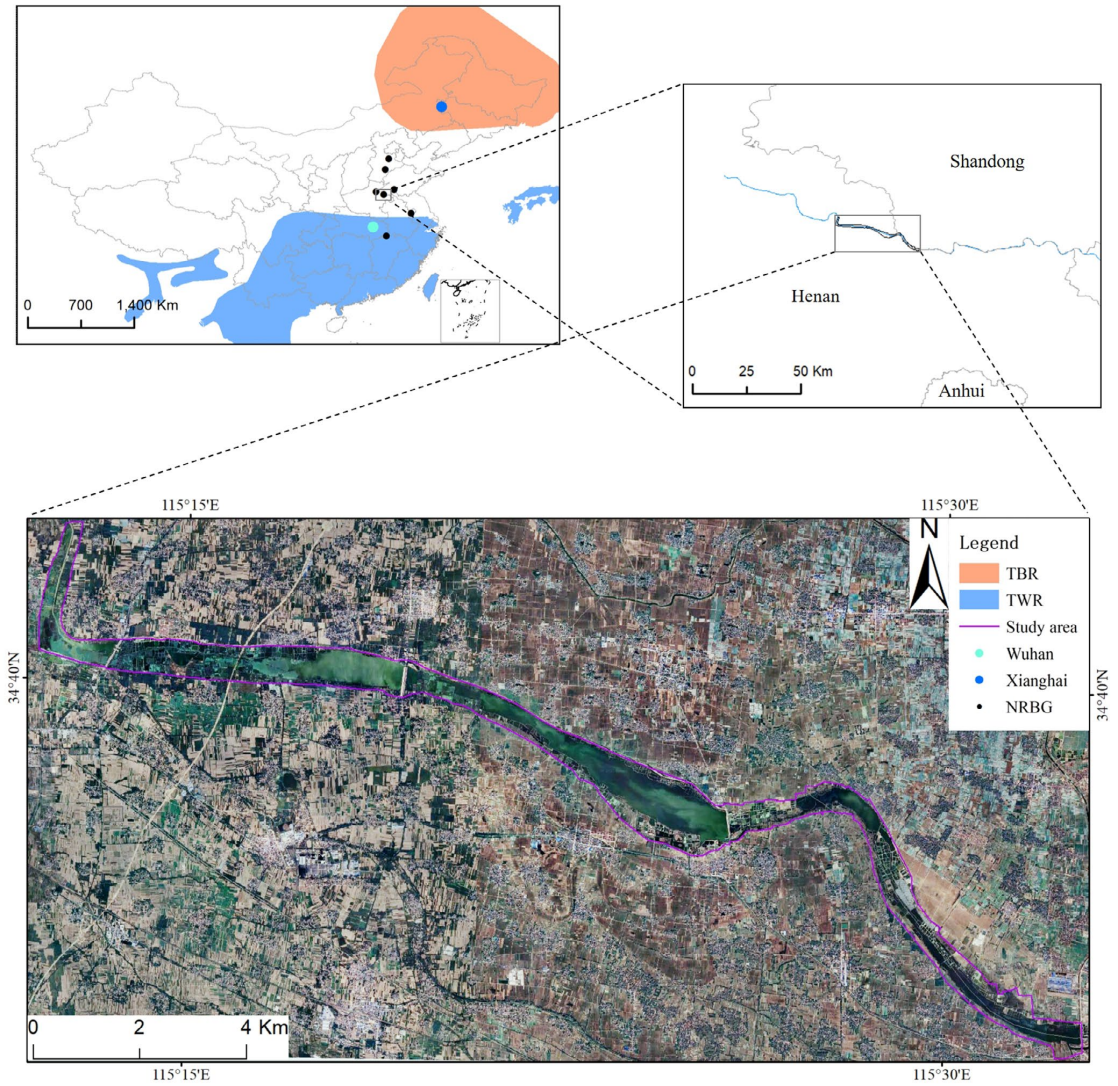
Map of the study area, the Ancient Yellow River National Wetland Park of Henan province, China. Orange indicates traditional breeding range (TBR) and blue indicates traditional wintering range (TWR), data obtained from the International Union for Conservation of Nature (IUCN). The black dots represent the newly recorded breeding grounds (NRBG).

Field research was conducted at Minquan Yellow River Gudao Wetlands (34°40′ N 115°18′ E) in Henan province, located on the North China Plain. The study area generally contains reservoirs, fish ponds, emergent wetlands, and grassland (Figure 2) and is considered an important breeding site for the Baer's Pochard in 2019 and designated as a Ramsar Site in 2020 (Ramsar Sites Information Service 2020). Since 2018, fish farming has been strictly prohibited for water source protection. Most ponds have been abandoned, while some have been converted to lotus cultivation. According to our observation, dominant plant species include Shining Pondweed ( *Potamogeton lucens* ), Reedmace (*Typha orientalis*), Lotus ( *Nelumbo nucifera* ), and Common Reed ( *Phragmites australis* ). The mean annual temperature in the study area is 14°C, with an approximate frost‐free period of 213 days and an average annual rainfall of 674 mm.

### 2.2 Nest Searching and Monitoring

**FIGURE 3:**
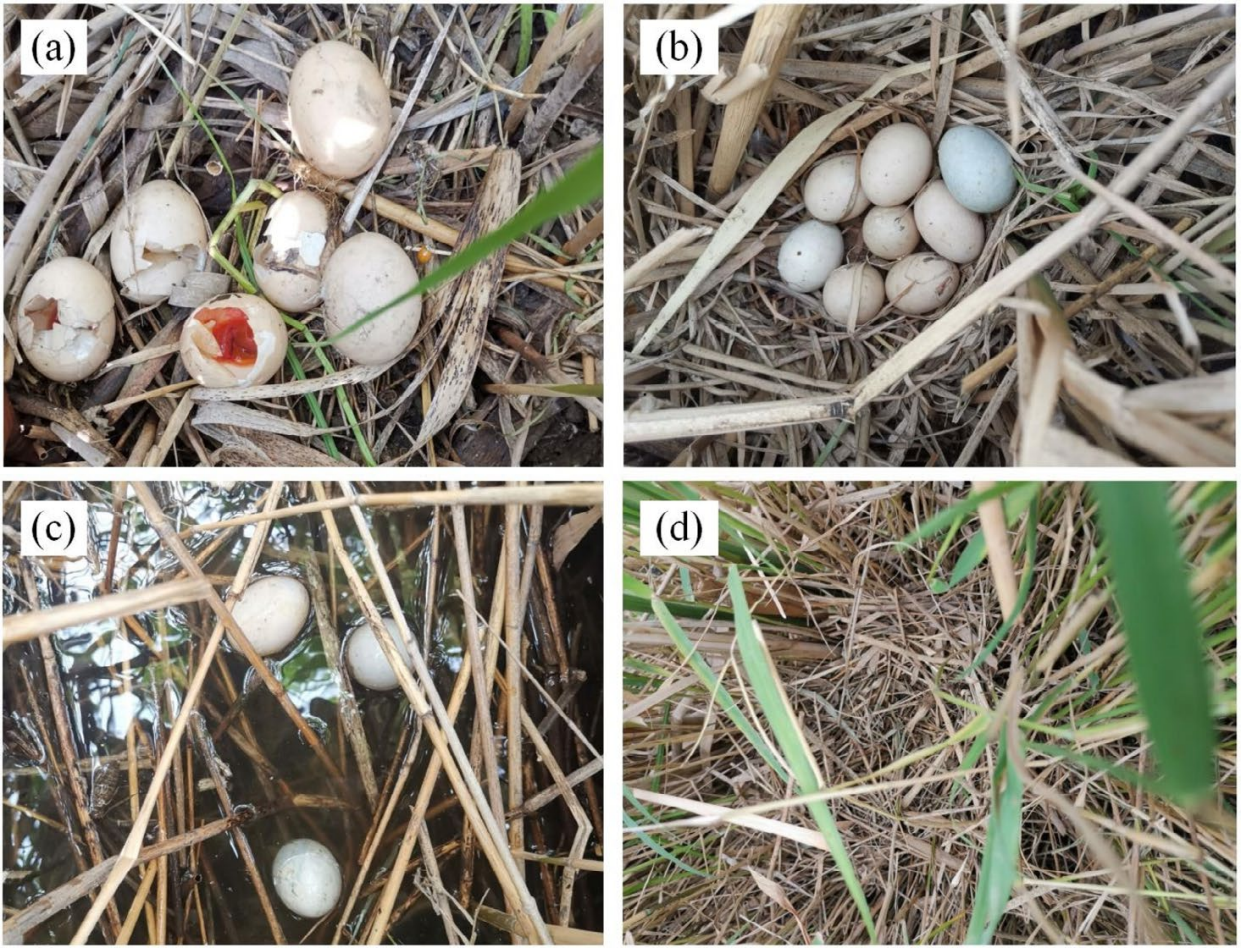
Images of various nest fates, predation (a), desertion (b), flooding (c), and egg collection (d).

Nest locations were determined by observing paired individuals and systematically surveying suitable breeding habitats from April to July during the years 2019–2022. Surveys were typically conducted during early morning and late afternoon to maximize detection probability. The surveyors waded through shallow wetlands, which are the main nesting habitats of the Baer's Pochard, to carry out the nest searching. Clutch size and incubation dates were determined through nest checks. Nests with more than one egg hatched were defined as successful (Klett et al. 1986), indicated by the presence of detached shell membranes, small egg fragments without membranes, and ducklings. Failed nests were classified as follows. Unattended nests with broken eggs were categorized as predation (Figure 3a), while those with intact eggs were classified as nest desertion (Figure 3b). Nests that were soaked in water were categorized as flooding (Figure 3c). If no eggs or egg shells were found in or near the nest, it was considered to be egg collecting by humans (Figure 3d).

### 2.3 Data Collection

The coordinates of each nest were recorded using a Global Positioning System unit. The date of the first egg laid was considered as the laying date (Lack 1950). For nests with ongoing incubation at discovery, eggs were candled to determine the incubation stage (Weller 1956) and estimate the laying date of the eggs. The mean clutch size was calculated using nests that completed egg laying. Egg length (L) and breadth (B) were measured with a digital vernier caliper (nearest 0.01 mm), and egg weight was recorded using an electronic scale (nearest 0.01 g). We calculated egg weight (W) using the formula *W = Kw* * *L* * *B* ^*2*^ as an alternative to initial weight, as bird eggs are known to lose weight after being laid. Kw is a species‐specific weight coefficient derived from values of W, L, and B (Hoyt 1979). The Kw of Baer's Pochard was calculated using only freshly laid eggs (≤ 1 day old). The Mayfield method was used to estimate nest success rate (Mayfield 1975). The hatch rate was calculated by dividing the number of hatched eggs by the clutch size.

For each nest, we measured thirteen habitat variables (Table 1). Distance to disturbance and mound area were estimated using Google Earth (version 7.3.4.8248). Mound formation time was determined by consulting land operators and browsing historical satellite images. Other environmental factors (mound width, distance to water, height above water, water depth, and vegetation height) were measured to the nearest 1 cm using a tapeline.

**TABLE 1:** Summary of predictor variables used to model nest success of Baer's Pochard ( *Aythya baeri* ), 2019–2022.

Variable	Units	Definition	Mean ± SD
Initial laying date (ID)	days	The day of one nest laying its first egg, day 1 is the earliest known nest initiation date for the entire study period (ordinal day 115, April 25).	34.06 ± 17.07
Mound length (ML)	m	Length of mounds have nest on.	33.60 ± 41.14
Mound width (MW)	m	Width of mounds have nest on.	1.56 ± 1.63
Mound area (MA)	m^2^	Area of mounds have nest on.	58.60 ± 115.65
Mound formation time (MFT)	year	Formation time of mounds have nest on.	7.28 ± 3.37
Distance to water (DW)	cm	Distance to the nearest water of nest.	49.20 ± 55.40
Height above water (HW)	cm	Vertical distance from water to nest bottom.	25.72 ± 30.07
Distance to disturb (DD)	m	Distance from nest to the nearest disturb activities.	29.40 ± 22.91
Water depth (WD)	cm	Depth of water around the nest mound.	42.28 ± 33.51
Distance to shore of mound (DSM)	m	Distance from nesting mound to nearest pond shore.	10.26 ± 7.95
Distance to shore of nest (DSN)	m	Distance from nest to nearest pond shore.	21.08 ± 17.54
Nest cover (NC)	%	The percentage of vegetation cover within a 50 × 50 cm sampling centered on the nest bowl (Lawson et al. 2021).	61.82 ± 19.03
Vegetation density (VD)	plants/dm^2^		24.14 ± 11.63
Vegetation height (VH)	m	Maximum height of vegetation directly over the nest	2.24 ± 0.81

### 2.4 Data Analysis

We used the logistic exposure method (Shaffer 2004) to estimate the DNS. This approach allows us to model the nest survival process while accounting for varying exposure times across nests. We used the Generalized Linear Mixed Model (GLMM) to estimate the relationship between DNS and environment variables. Samples that were anthropogenically failed or had indeterminate nest fate were removed from the analysis. We treated the study year as a random effect to control for unmeasured effects on nest success. The “dredge” function in the “MuMIn” package was used to construct the global model, and models were ranked using Akaike's Information Criterion (AICc) value. We select the best‐fitting model (with the smallest AICc) from the candidate model set as the top model. All the analyzes regarding the impact of habitat variables on DNS will be based on the top model. A variable was considered to have a significant effect when *p* < 0.05 for the estimated value of the variable. Multicollinearity tests were performed for all predictor variables using the “vif” function, with VIF < 10 considered to have low collinearity (Miles 2014) and suitable for further analysis. All statistical analyzes were performed using Program R (R version 3.6.1).

## 3 Results

In 2019, the breeding of the Baer's Pochard at the study site was recorded for the first time. Subsequent years of investigation indicated that the Baer's Pochard has a stable breeding population in Minquan. In our field survey, we recorded a total of 108 nests of Baer's Pochard, with 10, 28, 35, and 35 nests documented in the years through 2019 to 2022, respectively. Baer's Pochard exhibits a preference for nesting on well‐covered mounds and pond ridges. The majority of nests were located on mounds surrounded by water (*n* = 95), with others situated on broken pond ridges (*n* = 12) and pond ridges (*n* = 1). Females constructed their nests using surrounding vegetation. Initially, the nest bowl was simple in structure but became more refined throughout the egg‐laying process.

### 3.1 Laying Date

**FIGURE 4:**
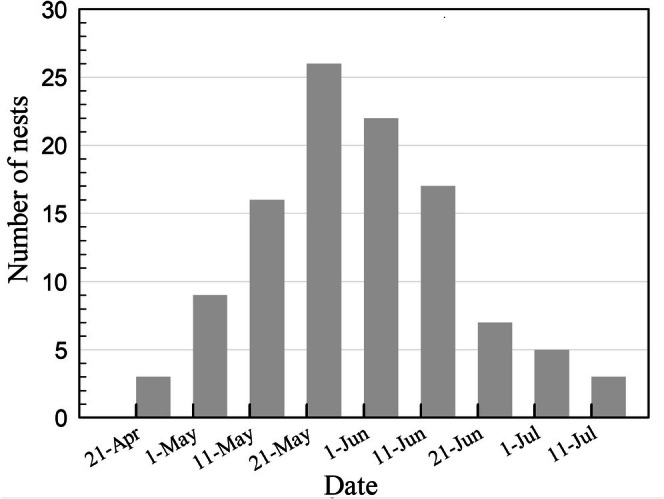
The number of nests that laid initial eggs on different dates (in ten‐day intervals). Notably, approximately 75% of all nests commenced egg laying between 10 May and 21 June.

Our findings indicate that the earliest recorded laying dates for Baer's Pochard were 29 April in 2020, 25 April in 2021, and 27 April in 2022, while the latest laying dates occurred on 15 July in 2020, 11 July in 2021, and 14 July in 2022. The average ± SD (same as below) duration of egg initiation within a given year was 79 ± 1 days, ranging from 78 to 80 days (Figure 4).

### 3.2 Measurements of Nests and Eggs

The dimensions of 31 completed nests were measured: outer diameter of 27.26 ± 3.48 cm (range: 22–37 cm), inner diameter of 16.03 ± 1.52 cm (range: 13–18 cm), nest height of 12.71 ± 2.82 cm (range: 7–20 cm), and nest depth of 9.08 ± 2.73 cm (range: 5–19 cm). Additionally, we measured the length and breadth of 296 eggs. The mean egg length was 52.81 ± 1.70 mm (range: 48.65–57.30 mm), and the mean egg breadth was 38.46 ± 1.16 mm (range: 34.27–40.74 mm). 31 eggs from 7 nests were laid within a single day at the time of measurement. This data was subsequently utilized to calculate the Kw of Baer's Pochard, yielding a value of 0.55. The average initial egg weight was 43.34 ± 3.38 g (range: 32.00–50.63 g, *N* = 296).

### 3.3 Clutch Size

Of the 57 nests that completed the egg‐laying, the mean clutch size was 12.79 ± 3.64 (range: 7–23). Notably, instances that eggs were laid after the females had commenced incubation and multiple eggs were laid in a single day were observed. We presumed these nests were parasitized by conspecifics. Among the 24 nests identified as parasitized, the mean clutch size was 15.83 (range: 11–23, *N* = 24). In contrast, non‐parasitized nests had a mean clutch size of 10.58 (range: 7–13, *N* = 33), which was significantly smaller than that of parasitized nests (independent two‐samples *t*‐test; *t* = −5.43, df = 35.67, *p* < 0.00001).

### 3.4 Nest Survival and Threats

We monitored a total of 103 out of 108 nests (10, 28, 32 and 33 nests in 2019, 2020, 2021 and 2022 respectively) until their nest fate was determined. Among the 103 nests whose fate could be determined, 24 nests successfully hatched at least one duckling.

The Mayfield method nest success rate was 24.97% based on 1551 exposure days (212 observations) from 103 nests. Predation was identified as the leading cause of nest failure, affecting 46 nests (44.66%). While a systematic predator survey was not conducted, infrared camera monitoring provided direct evidence of predation by the Siberian Weasel ( *Mustela sibirica* ) in two cases. Other factors contributing to nest failure included desertion, flooding, and egg collection (Table 2). Among the 24 successful nests, 21 experienced partial clutch loss, leading to 103 eggs failing to hatch. The average hatching rate for these nests was 73.11% (*N* = 24). We also compiled nest success rates for four additional *Aythya* species from seven studies. The values ranged from 21.00% to 61.00%, with a median of 50.00% (Table 3).

**TABLE 2:** Nesting success rates and nesting threats of the Baer's Pochard ( *Aythya baeri* ) in Minquan, Xianghai, and Wuhan, China.

Study area	Apparent nest success	Causes of nest failure (%)			
		Predation	Desertion	Flooding	Egg collecting
Minquan	23.30%	44.66	17.48	7.77	6.80
Xianghai	27.80%	16.67	44.44	0	11.11
Wuhan	37.77%	20.00	31.11	11.11	0

**TABLE 3:** Comparison of nest success rate (Mayfield method) between Baer's Pochard ( *Aythya baeri* ) and other *Aythya* species.

Species	Study area	Study period	Nest success rate	Sources
Baer's Pochard	C. China	2019–2022	24.97%	This study
	S. China	2018–2019	41.20%	Wei et al. (2020)
Canvasback ( *A. valisineria* )	W. USA	1970–1971, 1977–1985, 1998–2000	50.00%	Kruse et al. (2003)
Lesser Scaup ( *A. affinis* )	SW. USA	2006–2018	43.00%	Navarre et al. 2022
	NW. Canada	1991–1998	61.00%	(Fournier and Hines (2001))
Ring‐necked Duck ( *A. collaris* )	NE. USA	2008–2012	27.80%	Roy (2018)
Greater Scaup ( *A. marila* )	S. USA	1995	61.00%	Tatman et al. 2009
	S. USA	1996	21.00%	
	NW. Canada	1991–1998	58.00%	(Fournier and Hines (2001))

**FIGURE 5:**
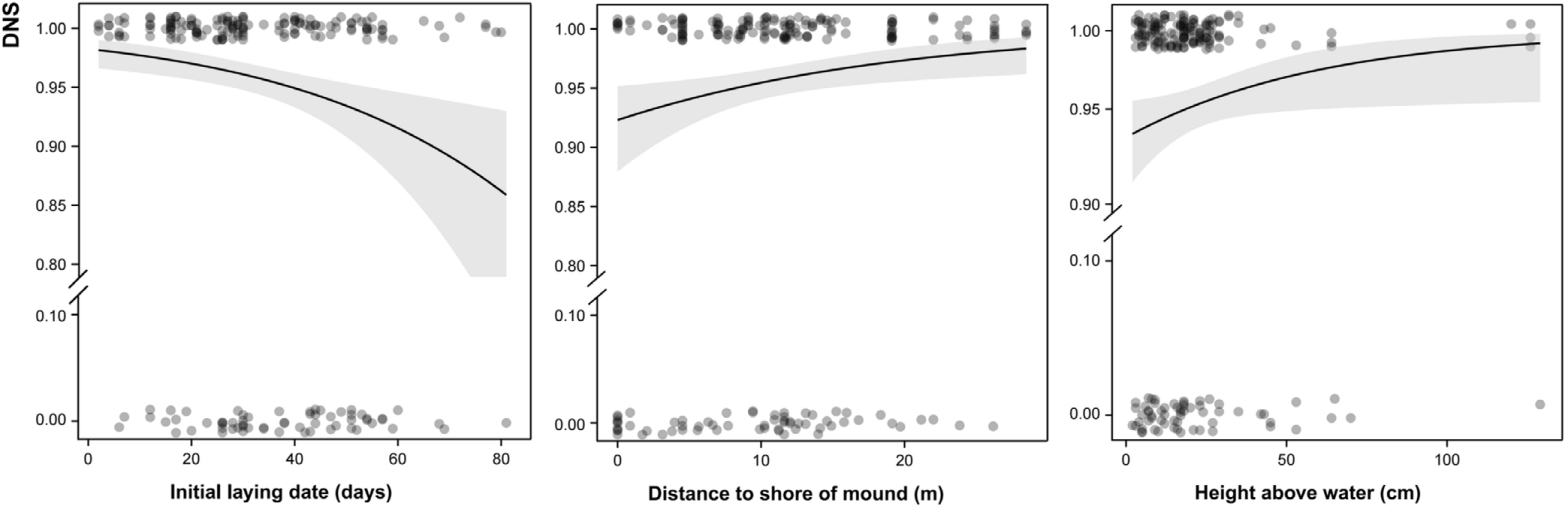
Predicted daily nest survival rate (with 95% CI) of Baer's Pochard ( *Aythya baeri* ) in relation to the initial laying date, distance to the shore of mound, and height above water.

We found no significant correlation or high multicollinearity among the environmental factors, so all factors were included in the analysis. The model selection results for nest survival based on AICc indicate that the model containing distance to shore of mound, height above water, initial laying date, and mound width is the most explanatory (Table 4; Table S1). Among the top‐ranked models, the distance to shore of mound and initial laying date were present in all models, while height above water, mound width, and water depth appeared in most models (Tables S1 and S2), suggesting that the relationships between nest survival and environmental factors are generally consistent across the models. The top model suggests the DNS have a negative relationship with increasing initial laying date (*β* = −0.027, *p* = 0.0009) and a positive relationship with increasing distance to shore of mound (*β* = 0.056, *p* = 0.0100) and height above water (*β* = 0.018, *p* = 0.0382; Figure 5).

**TABLE 4:** Model results for the factors that affect the nest survival of Baer's Pochard ( *Aythya baeri* ) breeding in the Ancient Yellow River National Wetland Park of HeNan province, China, 2019–2022.

Covariates^a^	Estimates	SE	*z*	*p* ^b^
Intercept	3.358939	0.387210	8.675	< 0.001***
DSM	0.056072	0.021781	2.574	0.010044*
HW	0.017745	0.008563	2.072	0.038247*
ID	−0.027402	0.008240	−3.325	0.000883***
MW	−0.246146	0.137195	−1.794	0.072792 .

^a^ Variable abbreviations: DSM = Distance to shore of mound, HW = Height above water, ID = Initial laying date, MW = Mound width.

^b^ Signif. codes: 0.001 ‘**’ 0.01 ‘*’ 0.05 ‘.’ 0.1 ‘’.

## 4 Discussion

The newly documented breeding site in Minquan extends the known breeding distribution of Baer's Pochard and updates conservation priority area. We conducted a quantitative assessment of the reproductive parameters and identified several nest threats of the critically endangered Baer's Pochard. Furthermore, we confirmed nesting time and two habitat variables significantly influenced daily nest survival of Baer's Pochard for the first time. Finally, our study provided evidence‐based recommendations to improve their breeding success, which are crucial for ensuring the species' long‐term survival.

### 4.1 Laying Date

In our study area, the Baer's Pochard started laying eggs in late April and end in mid‐July. Compared with the traditional breeding sites at higher latitudes, where the breeding period spans May to June (Gao et al. 1992), our observations indicate an earlier start time and a later end time. However, the breeding period at Wuhan, a lower‐latitude site, was similar to our findings (Wei et al. 2020). The plasticity of breeding time is important for birds to adapt to climatic variability (Dunn and Winkler 2010), especially for bird species breeding at high latitudes, where they need to complete incubation and chick‐rearing within a relatively short breeding season (Messmer et al. 2021). Recently, increasing recordings of Baer's Pochard breeding in central China have been documented. Our result, together with that in Wuhan (Wei et al. 2020), imply a potential longer breeding period in these areas, which may enhance reproductive output and aid population recovery.

Breeding periods vary widely among duck species in the northern Hemisphere, with average nesting dates differing by up to 60 days (Raquel et al. 2016). The Eastern Spot‐Billed Duck ( *Anas zonorhyncha* ), another species breeding at our study site, began laying eggs in mid‐March in our survey, obviously earlier than Baer's Pochard. This phenomenon has also been observed in the traditional breeding ground of Baer's Pochard (Gao et al. 1992). The differences in breeding timing are likely influenced by factors such as food availability, which has been shown to link with fledging period in many species (Both 2010). For example, a study on Lesser Scaup ( *A. affinis* ) has indicated that delayed nesting can benefit ducklings by providing them with more abundant and high‐quality food resources, which increase as the season progresses, thus increasing survival rates of the ducklings (Dawson and Clark 1996).

### 4.2 Nest Survival

In our study, the Mayfield nest success rate of the Baer's Pochard was 24.97%, which was lower than that in Wuhan (41.20%) and other *Aythya* species. This might indicate that the reproductive success rate is an important factor contributing to the population decline of the Baer's Pochard and underscores the urgency of implementing targeted conservation actions.

Understanding the relationships between spatio‐temporal factors and nest success of Baer's Pochard is essential for informing conservation practice. In our research, daily nest survival demonstrated a negative correlation with the laying date. This phenomenon is primarily explained by the notion that early breeding birds tend to occupy more favorable nesting habitat (Verhulst and Tinbergen 1991; Bloom et al. 2013). Moreover, seasonal degradation in habitat quality will contribute to a decline in nest success as the breeding season progresses (Verhulst and Nilsson 2008). For example, changes in predation intensity as the season progresses may contribute to temporary variations in nest success (Colwell et al. 2011), as nest predation is the main cause of nest failures in our research.

Our results also indicate a positive correlation between daily nest survival and the distance to the shore of the mound. We speculate that nests located further from the pond shore experience lower disturbance and predation risks. Predation pressure from land‐based predators has been found negatively correlated with water depth and distance from the edge in wetland systems because the ability of mammalian predators to access nests is weakened by water (Batáry and Báldi 2004; Albrecht et al. 2006; Schmidt et al. 2023). Previous studies on Mallards ( *A. platyrhynchos* ), Blue‐winged Teals (*Spatula discors*), Ring‐necked Ducks ( *A. collaris* ), and Lesser Scaups have also found that breeding on islands leads to higher daily nest survival due to reduced predation pressure (Townsend 1966; Fournier and Hines 2001).

Flooding is also a common cause of nest failure in ground‐nesting waterbirds (Warren et al. 2013; Elas et al. 2023; Thompson et al. 2023), especially in diving duck species that prefer nesting close to water (McAuley and Longcore 1989). More importantly, flooding as extreme natural events has increased because of climate change (Dankers and Feyen 2008). The positive relationship between height above water and daily nest survival is consistent with a study on other diving ducks, which reveals that the vertical distance to water positively affects nest success (Walker et al. 2005). Studies on other species show that breeding individuals may adjust nesting time to avoid extreme event periods and/or choose higher places to nest in the beginning of nest site selection to prevent the occurrence of nest flooding (Bailey et al. 2017). In addition to taking such prior avoidance strategies, we also observed a case in which a female Baer's Pochard heightened its nest by approximately 15 cm when facing flood risk. The similar phenomenon has also been documented in the Wuhan population (Wei et al. 2020), suggesting that heightening nests may be Baer's Pochard's strategy to cope with flooding risk.

### 4.3 Management Implications and Recommendations

Predation is the leading threat to nest survival for Baer's Pochard. High predation pressure may result from human activities, particularly a history of fish farming in our study site. Studies on other duck species have revealed that brood success is higher in forest lakes than that in agricultural landscapes (Gunnarsson and Elmberg 2008; Pieron and Rohwer 2010; Holopainen et al. 2024), indicating difference in local predator communities influenced by human activities (Rodewald et al. 2011; Borgo and Conover 2016; Lawson et al. 2021). To decrease predation pressure that beyond natural levels, implementing immediate actions such as predator management and the development of suitable nesting sites are crucial. Strategies such as predator removal and exclosure have been proven effective in improving nest success for upland nesting ducks (Pieron and Rohwer 2010; Gautschi et al. 2024; Gómez‐Silva et al. 2024). Additionally, providing upland nesting cover has emerged as common approach to avoid nest predation in regions such as the Prairie Pothole Region (Reynolds et al. 2001). Notably, while infrared camera trapping has documented Siberian Weasel acting as predators, more comprehensive surveys of predator identity and diversity are still needed in the future to inform evidence‐based predator management strategies.

The higher likelihood of success for earlier nests may indicate a lack of suitable nest sites in our study area. Evidence suggests that constructing appropriate nesting sites can significantly enhance nesting success (Kacergyte et al. 2021). The significant effect of distance to shore of mound on nest survival highlights the importance of creating nesting mounds positioned farther from the shore as a conservation strategy. Besides, Baer's Pochard exhibits a preference for nesting near water, making them vulnerable to both flooding and drought conditions. Dry conditions may influence nesting effort and expose nests to an increased range of predators (Smith et al. 2010; Howerter et al. 2014; Jones et al. 2024). Fluctuations in water levels due to farming activities also pose serious threats to nest survival. In Xianghai and Wuhan, nest desertion accounted for the majority of nest failures (Gao et al. 1992; Wei et al. 2020). This indicates that maintaining an appropriate water level during the breeding period has a positive effect on increasing nest survival.

Although nest survival is widely regarded as a critical demographic parameter for a population to remain stable or increase in many duck species (Jehle et al. 2004; Howerter et al. 2014). Other parameters such as the survival rate of fledglings, the expected lifespan of adult birds, and their survival rates have also influence population dynamics (Hoekman et al. 2002). However, the Baer's Pochard remains a species that has been rarely studied. Future research on demographic parameters is needed to better understand and protect this species. Besides, human activities, both direct (e.g., egg collection, aquaculture, and grazing) and indirect (e.g., influence on local predator community, landscape alteration), have significant impacts on nest success (Cheng and Ma 2023; Pagnon et al. 2024). Due to the absence of systematically collected data on human disturbance levels, a quantitative analysis of their impact was not feasible. We recommend that future research undertake quantitative assessments of human disturbance intensity. This will allow for a precise evaluation of the impact of anthropogenic pressure on nest fate, facilitating the development of targeted conservation strategies.

## Author Contributions

**Fuguang Ma:** investigation (lead), methodology (equal), software (lead), visualization (equal), writing – original draft (lead). **Yuanxing Ye:** project administration (equal), supervision (equal), writing – review and editing (lead). **Lu Li:** investigation (equal), methodology (equal). **Siyu Geng:** investigation (equal), writing – original draft (equal). **Zhongxin Li:** methodology (equal), project administration (equal). **Jianying Ren:** formal analysis (equal), methodology (equal), project administration (equal). **Changqing Ding:** funding acquisition (lead), methodology (equal), project administration (lead), supervision (equal), writing – review and editing (lead).

## Funding

This work was supported by National Natural Science Foundation of China (32270550), Society of Entrepreneurs & Ecology.

## Conflicts of Interest

The authors declare no conflicts of interest.

## Supporting information


**Appendix S1:** ece373181‐sup‐0001‐Appendix1.docx.

**Appendix S2:** ece373181‐sup‐0002‐Appendix2.xlsx.

## Data Availability

The data is provided as Supporting Information.
