# Supplementary material for: Breeding Ecology of the Critically Endangered Baer's Pochard (Aythya baeri): Nest Threats and Conservation Implications
Source: Ecol Evol. 2026 Feb 27;16(3):e73181. doi: 10.1002/ece3.73181 (PMC12949339; doi:10.1002/ece3.73181)
Supplement: Supplementary file 1 — Appendix S1: ece373181‐sup‐0001‐Appendix1.docx. [file ECE3-16-e73181-s001.docx]

**Appendix**

**Table S1.** Ranking of models explaining variation in nest survival of Baer's Pochard (*Aythya baeri*) breeding in the Ancient Yellow River National Wetland Park of HeNan province, China, 2019 – 2022.

| **Model structure ^a^** | **df** | **logLik ^b^** | **ΔAICc ^c^** | **Weight ^d^** |
| --- | --- | --- | --- | --- |
| DSM+HW+ID+MW | 6 | -167.02 | 0.00 | 0.06 |
| DSM+HW+ID+MW+NC+VH+WD | 9 | -163.87 | 0.18 | 0.06 |
| DSM+ID+NC+VH+WD | 7 | -166.12 | 0.35 | 0.05 |
| DSM+HW+ID+MW+WD | 7 | -166.16 | 0.43 | 0.05 |
| ID+NC+VH+WD | 6 | -167.32 | 0.60 | 0.04 |
| DSN+ID+NC+VH+WD | 7 | -166.35 | 0.81 | 0.04 |
| DSM+ID+WD | 5 | -168.57 | 0.99 | 0.04 |
| DSM+HW+ID+MW+NC | 7 | -166.44 | 0.99 | 0.04 |
| DSM+HW+ID+MW+NC+WD | 8 | -165.41 | 1.09 | 0.04 |
| DSM+HW+ID | 5 | -168.63 | 1.11 | 0.03 |
| DSM+HW+ID+MW+VH+WD | 8 | -165.49 | 1.25 | 0.03 |
| DSM+ID+NC+WD | 6 | -167.66 | 1.29 | 0.03 |
| DSM+HW+ID+MW+VH | 7 | -166.59 | 1.30 | 0.03 |
| DSM+HW+ID+MW+NC+VH | 8 | -165.52 | 1.31 | 0.03 |
| DSM+ID | 4 | -169.82 | 1.40 | 0.03 |
| DSM+ID+MW+NC+VH+WD | 8 | -165.64 | 1.55 | 0.03 |
| HW+ID+MW+NC+VH+WD | 8 | -165.64 | 1.56 | 0.03 |
| DSM+HW+ID+NC+VH+WD | 8 | -165.64 | 1.56 | 0.03 |
| DSN+ID+MA+NC+VH+WD | 8 | -165.68 | 1.62 | 0.03 |
| DD+DSM+HW+ID+MW | 7 | -166.77 | 1.65 | 0.03 |
| DSM+DW+HW+ID+NC+VH+WD | 9 | -164.62 | 1.68 | 0.03 |
| DSM+ID+VH+WD | 6 | -167.91 | 1.78 | 0.02 |
| DSM+DW+HW+ID+MW+NC+VH+WD | 10 | -163.57 | 1.79 | 0.02 |
| DSN+DSM+ID+NC+VH+WD | 8 | -165.77 | 1.81 | 0.02 |
| DSN+DSM+HW+ID+MW | 7 | -166.87 | 1.85 | 0.02 |
| DSM+HW+ID+NC | 6 | -167.95 | 1.87 | 0.02 |
| DD+ID+NC+VH+WD | 7 | -166.88 | 1.88 | 0.02 |
| DSM+DW+HW+ID+MW | 7 | -166.90 | 1.90 | 0.02 |
| HW+ID+NC+VH+WD | 7 | -166.90 | 1.92 | 0.02 |
| DSM+HW+ID+NC+VH | 7 | -166.92 | 1.95 | 0.02 |
| DSM+HW+ID+MW+VD | 7 | -166.93 | 1.98 | 0.02 |

^a^ Variable abbreviations: DSM = Distance to shore of mound; DSN = Distance to shore of nest; DW = Distance to water; HW = Height above water; DD = Distance to disturb; ID = Initial laying date; MW = Mound width; MA = Mound area; NC = Nest cover; WD = Water depth; VH = Vegetation height; VD = Vegetation density.

^b^ logLik is the maximized log-likelihood value

^c^ ΔAICc is the increase in AICc over the lowest observed value of AICc and the lowest AICc value was 346.44, Only models with ΔAICc ≤ 2 are shown.

^d^ Weights indicate the likelihood of a given model.

**Table S2.** Results of model 2 – 4 predicting daily nest survival of Baer’s Pochard (*Aythya baeri*) breeding in the Ancient Yellow River National Wetland Park of HeNan province, China, 2019 – 2022.

| **Covariates ^a^** | **Estimates** | **SE** | **z** | ***P* ^b^** |
| --- | --- | --- | --- | --- |
| Model 2 |  |  |  |  |
| (Intercept) | 3.503434 | 0.746503 | 4.693 | < 0.001 *** |
| ID | -0.032254 | 0.008572 | -3.763 | 0.0002 *** |
| MW | -0.258187 | 0.136773 | -1.888 | 0.0591 . |
| DSM | 0.040090 | 0.022901 | 1.751 | 0.0800 . |
| HW | 0.014502 | 0.008686 | 1.670 | 0.0950 . |
| WD | 0.010679 | 0.006500 | 1.643 | 0.1004 |
| VH | 0.386723 | 0.234406 | 1.650 | 0.0990 . |
| NC | -0.016319 | 0.009397 | -1.737 | 0.0825 . |
| Model 3 |  |  |  |  |
| (Intercept) | 3.500880 | 0.746175 | 4.692 | < 0.001 *** |
| ID | -0.030514 | 0.008272 | -3.689 | 0.0002 *** |
| DSM | 0.032196 | 0.022206 | 1.450 | 0.1471 |
| WD | 0.011220 | 0.006505 | 1.725 | 0.0846 . |
| VH | 0.383990 | 0.232433 | 1.652 | 0.0986 . |
| NC | -0.016750 | 0.009221 | -1.817 | 0.0693 . |
| Model 4 |  |  |  |  |
| (Intercept) | 3.247492 | 0.404337 | 8.032 | < 0.001 *** |
| ID | -0.029341 | 0.008580 | -3.420 | 0.0006 *** |
| MW | -0.262949 | 0.137858 | -1.907 | 0.0565 . |
| DSM | 0.052250 | 0.022104 | 2.364 | 0.0181 * |
| HW | 0.015456 | 0.008798 | 1.757 | 0.0790 . |
| WD | 0.007366 | 0.006184 | 1.191 | 0.2337 |

^a^ Variable abbreviations: ID = Initial laying date; MW = Mound width; DSM = Distance to shore of mound; HW = Height above water; WD = Water depth; VH = Vegetation height; NC = Nest cover.

^b^ Signif. codes: 0.001 ‘**’ 0.01 ‘*’ 0.05 ‘.’ 0.1 ‘ ’
